# Explaining the consequences of missed nursing care from the perspective of nurses: a qualitative descriptive study in Iran

**DOI:** 10.1186/s12912-022-00839-9

**Published:** 2022-03-14

**Authors:** Maryam Janatolmakan, Alireza Khatony

**Affiliations:** 1 Social Development and Health Promotion Research Center, Health Institute, Kermanshah University of Medical Sciences, Kermanshah, Iran; 2 Infectious Diseases Research Center, Kermanshah University of Medical Sciences, Kermanshah, Iran

**Keywords:** Missed nursing care, Nurse, Outcome, Qualitative research

## Abstract

**Background:**

Missed nursing care is a global challenge that can have many consequences. Knowing the experiences of clinical nurses can be helpful. Therefore, this study was conducted to explain the experiences of Iranian nurses regarding the consequences of missed nursing care.

**Methods:**

This qualitative descriptive study was conducted with a content analysis approach. Sampling was done by the purposeful sampling method and continued until data saturation. Data were collected by in-depth semi-structured interviews. Data were analyzed using qualitative content analysis and Graneheim and Lundman’s method. MAXQDA version 10 software was used for data management.

**Results:**

The participants included 14 nurses with a mean age of 38.7 ± 7.7 years. The data were classified into three categories: patient-related outcomes, nurse-related outcomes, and organization-related outcomes. These categories included nine subcategories entitled "moral distress", "job dissatisfaction", " decreased quality of nursing care "," patient dissatisfaction ","adverse events"," absenteeism ","intention to leave and subsequent turnover", "decreased hospital credit", and "increased hospital costs".

**Conclusion:**

Missed nursing care can have adverse consequences for the patients, nurses, and organizations. Therefore, it is necessary to adopt management strategies such as providing sufficient manpower and increasing nurses' job satisfaction to reduce the amount of missed nursing care. Further studies are needed to explain the predictors of the missed nursing care consequences.

## Introduction

Missed nursing care is a global challenge that poses a threat to the patient's safety and health [1, 2]. Missed nursing care includes nursing care not provided at all, incompletely performed, or delayed [1]. The rate of missed nursing care in the world varies from 10 to 50% [3–5]. Some of the most common types of missed nursing care, from the nurses' point of view, include lack of oral care, failure to observe proper medication time, lack of skincare, and lack of attention to the patient's religious needs [4, 6–12]. Numerous factors are associated with the occurrence of missed nursing care, including mismanagement, lack of financial and human resources, lack of interpersonal communication, weak teamwork, and high workload of nurses [8, 13–17]. Evidence suggests that missed nursing care has several negative consequences, including decreased patient and nurse satisfaction, willingness to leave, absenteeism, and increased length of hospital stay [3, 6, 7, 9, 10, 18–24].

Studies on the consequences of missed nursing care are very limited and mostly quantitative [19, 25, 26]. These outcomes can vary in different clinical and organizational contexts, and knowing the experiences of nurses involved in nursing care can be useful and constructive. Therefore, this qualitative study was conducted to explain the experiences of Iranian nurses regarding the consequences of missed nursing care. The results of this study can be used by healthcare policymakers to take the necessary measures to manage the adverse outcomes of this type of care.

## Materials and methods

### Study design

This descriptive qualitative study was done with an inductive content analysis approach. Content analysis refers to any technique that is used to systematically and objectively deduce the specific features of messages [27]. In content analysis, two basic deductive reasoning and inductive reasoning are used. Inductive reasoning moves from part to whole and from empirical data to theory, but deductive reasoning moves from the whole to the part and from theory to experience and observation [28, 29]. In inductive reasoning, one can discover the rules governing specific areas by observing and thinking carefully about repetitive patterns [30, 31].

### Participants and sampling method

This study was performed in one of the subspecialty hospitals of Kermanshah-a western province of Iran. The study was started with purposive sampling and continued until data saturation. The criteria for selecting the participants were having a bachelor's degree or higher in nursing, having at least one year of clinical experience, and willingness to participate in the study. The sample size was determined based on data saturation. Data saturation means that no new information is obtained from the continuation of the interviews [31, 32]. In this study, the data were saturated in the fourteenth interview.

### Study tools

In-depth, face-to-face, and semi-structured interviews were used to collect data. In addition to the interviews, field notes were used. Guide questions were used to direct the interviews, such as "Please describe your experiences with missed nursing care ", "What are the consequences of missed nursing care for patients? ", "What are the consequences of missed nursing care for the health centers?, and "What are the consequences of missed nursing care for nurses?". In addition, exploratory phrases such as "please explain more", "Why?", and “How?" were used to clarify the concepts.

### Data collection method

To select the participants, the researcher referred to different wards of the hospital and selected the participants. In the next step, the place and time of the interviews were determined with the participants’ agreement. The total number of interviews was fourteen. The duration of each interview was between 40 and 50 min. All interviews were conducted in the second author's office, which was located in a quiet area. All interviews were conducted by the first author, a professor with PhD. in nursing and an expert in qualitative research. It should be noted that all interviews were conducted in one step.

### Data analysis

Simultaneously with data collection, data were analyzed using conventional content analysis and based on Graneheim and Lundman’s method. Each interview was transcribed verbatim and then reviewed several times to gain a general understanding of the participants' statements. In the next step, according to the purpose of the study, the meaning units were identified, and appropriate codes were written for each meaning unit. The primary codes were categorized and named based on conceptual similarity, and subcategories were developed. In the next step, the subcategories were compared, and categories were formed. MAXQA software (version 10) was used to manage the data.

### Trustworthiness

Lincoln & Guba’s criteria, including credibility, transferability, dependability, and confirmability were used to evaluate the quality of the data [33–35]. To increase the credibility of the data, strategies such as long engagement, peer check, and member check were used. In order to increase the transferability of the data, the research results were provided to three nurses, and the degree of conformity of the results with their experiences was examined and confirmed. In order to increase the dependability, all stages of the research were described step by step so that a correct judgment could be made about it during evaluation by other people. To increase the confirmability of the data, parts of the interviews along with their analyses were provided to two external observers who were proficient in qualitative research as well as the subject under study, following which the coding accuracy was confirmed.

### Ethical considerations

This study is part of a research project (with number 980864) that has been approved by the ethics committee of Kermanshah University of Medical Sciences, with the code IR.KUMS.REC.1398.871. At the beginning of the interviews, the objectives of the research and confidentiality of personal information were explained to the participants. Informed written consent was obtained from all participants.

## Results

The mean age of the participants was 38.7 ± 7.7 years. Half of them were female (*n* = 7) and single (*n* = 7). Most participants had the position of nurse (*n* = 8) and bachelor's degree (*n* = 8) (Table 1).

**Table 1:** Demographic characteristic of participants

Variables		N (%)
**Sex**	Female	7 (50.0)
	Male	7(50.0)
Age (mean, SD^*^)		38.7 ± 7.7
**Marital status**	Married	7(50.0)
	Single	7 (50.0)
**Academic degree**	Bachelor of science	8(57.1)
	Master of science	6(42.9)
**Job title**	Nurse	8(57.1)
	Staff nurse	3(21.4)
	Head nurse	3(21.4)
**Job experience, year (mean, SD)**		10.1 ± 6.6

Standard deviation

Analysis of data on nurses' experiences about the consequences of missed nursing care yielded nine subcategories, including moral distress, job dissatisfaction, decreased quality of nursing care, patient dissatisfaction, intention to leave and subsequent turnover, absenteeism, decreased hospital credit, and increased hospital costs. These sub-categories were expressed in three categories, including "nurse-related consequences", "patient-related consequences", and "organization-related consequences". These categories identified a common theme called "consequences of missed nursing care" (Table 2).

**Table 2:** Categories and subcategories related to the nurses’ perspectives on the consequences of missed nursing care

Theme	Categories	Subcategories
Consequences of missed nursing care	Nurse-related consequences	Moral distress
		Job dissatisfaction
	Patient-related consequences	Decreased quality of nursing care
		Adverse events
		Patient dissatisfaction
	Organization-related consequences	Absenteeism
		Intention to leave and subsequent turnover
		Decreased hospital credit
		Increased hospital costs

## Nurse-related consequences

### Moral distress

The participants believed that when a nurse is engaged in an activity that is contrary to her moral teachings, she becomes morally distressed. In this regard, one of the participants stated:

"A nurse who does not provide the necessary training to her patient or does not pay attention to her communication needs acts exactly against the principles of professional ethics she has been taught, which causes moral stress and turmoil" (P. 8).

Another participant said:

"A nurse who should monitor the patient's vital signs during the blood transfusion every half an hour will suffer from moral distress and tension if she fails to do so, certainly because of this negligence" (P.5).

### Job dissatisfaction

Job satisfaction is an important part of nurses' lives. The participants believed that there was a close relationship between missed nursing care and nurses’ job satisfaction and stressed that patient dissatisfaction could affect their job satisfaction. One participant, while confirming this issue, stated:

"When the patient does not receive all the care he/she needs or receives it late, he/she experiences dissatisfaction, which can be transferred to the nurse through his/her behavior and speech. This patient dissatisfaction makes the nurse feel that she has not done her job well" (P.12).

Another participant said:

“A nurse who, despite her inner desire, does not have the opportunity to care for the patient has a negative view of her job and feels that she has not done her job properly” (P. 11).

## Patient-related consequences

### Decreased quality of nursing care

The participants believed that the main consequence of missed nursing care was a reduction in the quality of nursing care provided to the patients. They cited consequences such as endangered patient safety, prolonged hospital stay, and even death. One of the participants confirmed this and said:

"Failure to pay attention to the oral health of the intubated patient prolongs the process of extubation and separation of the patient from the ventilator, and as a result, the patient stays more in the ICU" (P.12).

Another participant said:

"Some nurses are not very careful about prescribing blood products and care during and after transfusions, which can be dangerous and even cause the death of the patient" (P.13).

### Patient dissatisfaction

All participants emphasized that the main goal of the healthcare system is to satisfy patients, and missed nursing care causes patient dissatisfaction. One of the participants confirmed this issue and said:

" When the nurse does not pay attention to relieving the patient's pain and defecation needs in time, the patient will be discharged with dissatisfaction" (P. 1).

Another participant stated:

"I had a patient whose angiocatheter was not fixed well by a nurse, and the chemotherapy drug had gone under his skin, so he had necrosis. This patient will be dissatisfied with the hospital services" (P. 4).

## Organization-related consequences

### Absenteeism

The participants reported excessive work pressure as one of the main reasons for nurses' absence from work. In this regard, one of the participants said:

"Now with the outbreak of Covid-19, the hospital is facing a shortage of nurses, which puts a lot of pressure on nurses, and they cannot provide all the care to patients. Sometimes the workload is so high that nurses will be absent from work" (P.6).

Another participant stated:

"The workload is sometimes so great that the nurse is forced to leave work for unrealistic reasons such as a cold or her child's illness" (P.7).

### Intention to leave and subsequent turnover

Lack of nurses is closely related to missed nursing care. All participants emphasized that the shortage of nurses puts additional work pressure on nurses and increases their intention to leave their job or undergo turnover. This has adverse consequences for the organization, such as loss of human capital, reduced productivity, and increased hospital costs. One of the participants said:

"In the current situation, nurses are under a lot of pressure from patients and the management system, and expectations from them are irrationally high. Nurses are not able to meet all these expectations, which causes a high rate of turnover or intention to leave the job "(P. 10).

Another participant, while emphasizing the costs of nurses' turnover or intention to leave, said:

“The nurses’ intention to leave leads to a shortage of nurses, thereby increasing the workload and burnout of the nursing staff. In addition, hospital productivity decreases and hospital costs increase” (P.14).

### Decreased hospital credit

The emphasis of each hospital management is on stakeholders' satisfaction and optimal service delivery. Any disruption in the provision of care leads to patient dissatisfaction and reduces the credibility of the hospital. In this regard, one of the participants, while confirming this issue, stated:

"When the expected patient care is not provided in a standard way, it leads to their dissatisfaction with the hospital and reduces the credibility of the hospital" (P.9).

Another participant stated:

"When, the patient has an accident such as falling from bed due to the negligence of a nurse, the position of the hospital is questioned by the clients as well as the whole community" (P.2).

### Increased hospital costs

The participants believed that missed nursing care could cause many hospital costs for providing human resources, facilities, and equipment. Emphasizing this issue, one of the participants said:

"The nurse's ignorance of the early signs of thrombophlebitis can cause thrombosis and its subsequent complications such as embolism, which in turn prolongs the length of hospitalization and imposes related costs such as providing medicine, nurses, etc. to the hospital" (P.3).

Another participant said:

"When a patient develops a bed sore due to the nurse's negligence, it increases the pressure on the system to provide manpower, facilities and equipment, paraclinical services, etc., all of which are costly" (P. 5).

## Discussion

This study aimed to explain the experiences of Iranian nurses regarding the consequences of missed nursing care. The participants believed that missed nursing care had several consequences, including moral distress, job dissatisfaction, decreased quality of nursing care, patient dissatisfaction, intention to leave and subsequent turnover, absenteeism, decreased hospital credit, and increased hospital costs. Nurses regarded moral distress as a consequence of missed nursing care. By definition, when a person knows what a moral act is but is unable to perform it for various reasons, he or she experiences moral distress [36]. Numerous factors play a role in moral distress, including the inability to cope with patients' critical situations [37], the feeling of inadequacy, and being between the two senses of ideal care and clinical reality [36]. Evidence suggests that there is a direct and two-way relationship between missed nursing care and moral distress. Moral distress can reduce nurses' ability to care for patients and prevent them from providing appropriate services to patients [25].

The participants believed that nurses’ satisfaction was low for various reasons, such as high workload, lack of manpower, and low salaries, and pointed out the role of nurses' dissatisfaction in missed nursing care. Evidence suggests that nurses’ satisfaction is directly related to patient satisfaction with the quality of nursing care [38]. When the nurse is not able to provide standard and quality care, job satisfaction is affected negatively [39]. The results of a study indicated that patient satisfaction is high in hospitals where nurses have high job satisfaction. In contrast, in hospitals where nurses have low job satisfaction, patient satisfaction is very low [38]. Considering the effect of nurses' dissatisfaction on the rate of missed nursing care, necessary measures should be taken to eliminate or moderate the causes of dissatisfaction in nurses.

The participants considered care the heart of nursing activities and the reduced quality of care an important consequence of missed nursing care. Evidence suggests that missed nursing care is associated with a decline in the quality of care [26]. Numerous factors affect the quality of care, including the use of qualified nurses, appropriate working conditions, and nurses’ high job satisfaction [40]. Therefore, to reduce missed nursing care and increase the quality of care, it is necessary to pay sufficient attention to the factors that promote care behaviors in nurses, especially to eliminate the factors involved in their dissatisfaction.

One of the consequences of missed nursing care is the increased risk of adverse events for patients, which was emphasized by all participants. In this regard, the results of a study indicated that missed nursing care could lead to various consequences such as nosocomial infections, pressure ulcers, falls, and medication errors, which ultimately increased hospital stay, readmission, and even death [26].

Numerous factors affect patients' satisfaction with health services, including the quality of nursing care. Evidence suggests that there is a close relationship between missed nursing care and patient dissatisfaction [18, 41], as patient satisfaction is higher in hospitals with better quality of clinical care and more nurses [18]. Therefore, providing suitable working conditions for nurses and gaining their satisfaction can be effective.

Nurses' working conditions can be so harmful that they cause a variety of physical and mental disorders and make them leave their work. Some of the factors related to absence from work are high workload, long working hours, and inappropriate interpersonal relationships, which are also among the causes of nurses’ dissatisfaction [42]. Previous studies have shown a close relationship between nurses' job satisfaction and missed nursing care and between job satisfaction and absenteeism. Thus, the rate of absenteeism is high in nurses with low job satisfaction [15, 43]. Therefore, to reduce the nurses’ absence from work, it is necessary to take specific actions to eliminate the causes of nurses' dissatisfaction.

Another consequence of missed nursing care is nurses' intention to leave and subsequent turnover. Although it is not clear how missed nursing care affects nurses' intention to leave, evidence suggests a relationship between missed nursing care and nurses’ intention to leave their position [19]. In a qualitative study, nurses cited the inability to provide standard care, or concerns about the possibility of making mistakes as reasons for intention to leave [44]. It seems it is possible to reduce nurses’ intention to leave and to provide quality nursing care by improving the working conditions and increasing the nurses’ satisfaction.

The participants believed that missed nursing care would damage the reputation of care centers by reducing their quality and safety. The results of a study by Aiken et al. (2021) indicated that patients' perceptions of the quality of care in medical centers were greatly impaired due to increased missed nursing care [45]. In this regard, an Iranian proverb says that a dissatisfied customer dissatisfies 13 people and a satisfied customer satisfies 3 people. Therefore, to maintain their status and credibility, the care centers should take the necessary measures to eliminate or reduce missed nursing care.

Complications associated with missed nursing care, such as pressure sores or falls, can lead to increased healthcare costs. In this regard, evidence suggests that the complications of missed nursing care in many cases increase the hospital stay and costs [8, 46–48]. The results of a study indicated that the cost of medication errors in the United States was more than $ 40 billion annually [49]. On the other hand, the occurrence of complications related to missed nursing care negatively influence the reputation and position of the hospital among patients and clients, which decreases the hospital income by reducing the number of clients [8].

### Study limitations

This study was conducted with a qualitative approach and therefore faces limitations in generalizing the findings.

## Conclusion

Missed nursing care is a global challenge that has different implications for patients, nurses, and organizations. The results showed that missed nursing care can cause patient dissatisfaction, various physical and psychological complications, and even death. The consequences of missed nursing care can also cause nurses’ moral distress and dissatisfaction. The organization-related consequences are increased costs, reduced organizational credibility, absenteeism, and intention to leave and subsequent turnover. Given the various consequences of missed nursing care, the healthcare centers need to increase nurses' job satisfaction and encourage them to report this type of care. Strengthening the monitoring systems can also be an important strategy to reduce missed nursing care. Further studies are recommended to investigate the consequences of missed nursing care and determine its predictors.

## Data Availability

The identified datasets analyzed during the current study are available from the corresponding author on reasonable request.
